# Construction Notes

**Published:** 1893-10-07

**Authors:** 


### CONSTRUCTION NOTES.

### New Charnwood Forest Convalescent Home.

The foundation stone of this building now in course of erection was laid by the Duchess of Rutland in the first week of August. The altitude of the site, which comprises an area of 450 feet, is above ordnance data. The plans of the architect (Mr. George H. Barrowcliff, of Loughborough) show a design comprising ground, first and second floors, with a spacious corridor running the entire length of each. The entrance hall, 27 feet by 16, will be available as a committee-room or for patients to receive their friends in and is divided from the men's and women's corridors by swing doors. The remainder of the front consists of three sitting-rooms, and a matron's-room. The back portion of the main building ground floor consists of dining hall, sitting-room, china and store cupboards, whilst the main staircases at either end lead to the men's and women's sleeping-rooms. At the rear are kitchen, scullery, larder, and other offices opening into a large paved yard, at the side of which a coach yard is being erected. The first floor is so arranged that the bed-rooms open direct into the main corridor and consists of bed and bath rooms; and by the arrangement of doors in the corridor a portion of the women's bed-rooms can be made available for the accommodation of men were this found to be necessary. The second floor on the women's side contains seven bed-rooms and cistern-room, with corridor. It is proposed to devote the whole of this floor to female patients. The ventilation generally has been carefully considered. The house is designed for 45 patients, and for the entire separation of the sexes, except at meal times. The sitting and bed rooms will be heated by open fire grates and the corridor and dining-room by water pipes. The cost of the structure complete, including purchase of land, water supply, furnishing, &c., will be about £6,000., and the contract is being carried out by Messrs. W. Moss and Son, of Loughborough.

